# Henry Hobart Keech

**Published:** 1884-04

**Authors:** 


### OBITUARY.

### IN MEMORIAM.

**At his residence in Baltimore, on Thursday, April 3d, 1884, after ten days' illness, of pneumonia, HENRY HOBART KEECH, M. D., D. D. S., aged fifty-three years.**

I feel not only embarrassed, but painfully solemn in attempting to cast a retrospective glance over the labors of such a man as Dr. Keech, and, to express the profound sense of the loss sustained by the profession that so warmly loved him, the denomination whose interests he so earnestly guarded and enlarged, and the community of which he was so valuable and useful a citizen.

It recalls years of labor, of constant courtesy, of kindly intercourse, of wise discretion, of manly qualities, of Christian graces that characterized his life and that won love and esteem; for he was a man beloved by all who knew him for his tenderness of heart and genial spirit, for his uprightness of conduct and consecration to his Master's work. We knew the simple beauty of his life; we knew its truth, its kindness, its helpfulness, its strength. Thus we knew and loved him, and thus we sorrow for him. He has preceded us to a better world to receive the reward of his labors.

Henry Hobart Keech, M. D., D. D. S., was born January 25th, 1831, in Harford County, Maryland, and was named after Bishop Hobart, of New York, an intimate friend of his father, Rev. John R. Keech, a minister of the Protestant Episcopal Church. His mother was the daughter of Judge John Scott. He spent his youth on a farm owned by his father, and received his earlier education partly from his father's instructions and partly from a district school. He entered the Baltimore College of Dental Surgery and after the usual course graduated in 1857. He engaged at once in dentistry and made it the uninterrupted business of his life. He practiced more than a year in several counties of Maryland, but July 1858, he removed to Baltimore City where he early acquired a reputation. His career as a dentist covers a period of more than a quarter of a century, and was one of unbroken, ever-growing popularity.

January 1860, Dr. Keech married Miss Hattie B. Pigman, who, with one daughter and one son survives him. Had he lived till January 25th, 1885, he would have celebrated his birthday and his silver wedding. Though Dr. Keech was for so many years absorbed in the duties of his profession and his church, yet it was in his home where he was eminently congenial and companionable that he was best known. There he manifested those domestic attachments and private virtues which characterized him as a devoted husband and father, and hence there he will be truly missed. His circumstances were those of entire worldly ease and comfort, and few men had a serener life. His nature took the utmost pleasure in giving pleasure to others, his charities were unostentatiously dispensed. At his bounteous board every guest however obscure and humble was made to feel perfectly at home.

In 1861, Dr. Keech was elected Demonstrator of Operative Dentistry in the Baltimore College of Dental Surgery. In 1873, he was appointed Professor of Anatomy in the Maryland Dental College, then organized. In 1874 he received the degree of M. D., from the Washington University, Baltimore. Subsequently for four years he occupied the chair of Dental Pathology and Therapeutics in the Maryland Dental College, which he resigned to devote more of his time and attention to mission work in which he had for some time been engaged. With two warmly-attached friends, Gen. George. H. Steuart and Mr. Eugene Poultney, as co-laborers, he took entire charge of a mission church now the church of the Holy Evangelists.

Dr. Keech published little; we are aware of nothing but some incidents of office-practice. Nevertheless he was a most indefatigable laborer and pre-eminently a man of system and method, governing himself even in the minutest particulars by exact rules. He was the instructor, guide, friend and idol of many young men who were successively under his care, to whom the result of a practical experience were furnished in a practical form; and he always remained among the truest the best, the most affectionate acquaintances with whom their life had been associated. He enjoyed an intimacy of relation and a fulness of confidence that made him a friend to whose wise judgment and loving sympathy no appeal from the difficulties and perplexities of life were made in vain.

The dental profession of which he was an ornament was prompt to recognize its loss. The dentists of Baltimore met and adopted a series of resolutions expressive of their high regard for the pure and useful life of the deceased and their appreciation of his long and able services.

R. G.

At a meeting of the dental profession of Baltimore, held at the office of T. S. Waters, Friday, April 4th, 1884, to take action in regard to the death of Dr. Henry Hobart Keech, the following was adopted:

*Whereas*, It has pleased Almighty God, in the administration of His Divine Providence, to call from among us by the hand of death our beloved friend and professional brother, Dr. Henry Hobart Keech, and,

*Whereas*, The professional, religious and social life of Dr. Keech was so pure and elevated in its tone and influence, that we desire to bear testimony to its excellence and hold it up for the emulation of others; therefore,

*Be it resolved*, That in the death of Dr. Keech the Dental Profession has lost one of its brightest ornaments—a man of talent, coupled with patient, industrious research and a conscientious performance of all the details of his work—one who by precept and example sought to raise the standard of his calling and fix it securely in the brotherhood of the learned professions; that the Church has lost a faithful and devoted follower of the Master; the community, a valuable, influential and benevolent citizen, and each of us especially, a genial, warm-hearted friend; that we extend to the bereaved family of the deceased our warmest sympathies in this the time of their sad affliction; that a copy of these resolutions be sent to the family of the deceased, and that they be published in the daily papers and dental journals.

M. W. FOSTER, M. D., D. D. S., *Chairman.*

*Committee.* { RICHARD GRADY, D. D. S.<br>T. S. WATERS, D. D. S.<br>B. HOLLY SMITH, JR. D. D. S.

